# Author Correction: Model-based assessment of Chikungunya and O’nyong-nyong virus circulation in Mali in a serological cross-reactivity context

**DOI:** 10.1038/s41467-023-36821-5

**Published:** 2023-05-03

**Authors:** Nathanaël Hozé, Issa Diarra, Abdoul Karim Sangaré, Boris Pastorino, Laura Pezzi, Bourèma Kouriba, Issaka Sagara, Abdoulaye Dabo, Abdoulaye Djimdé, Mahamadou Ali Thera, Ogobara K. Doumbo, Xavier de Lamballerie, Simon Cauchemez

**Affiliations:** 1 Mathematical Modelling of Infectious Diseases Unit, Institut Pasteur, Université de Paris, UMR2000, CNRS, Paris, France; 2 Unité des Virus Émergents, (UVE: Aix-Marseille Univ-IRD 190-INSERM 1207-IHU Méditerranée Infection), Marseille, France; 3 Malaria Research and Training Center, USTT, Bamako, Mali; 4 Centre d’Infectiologie Charles Mérieux, Bamako, Mali; 5 EA7310, Laboratoire de Virologie, Université de Corse-Inserm, Corte, France

**Keywords:** Statistical methods, Viral infection, Epidemiology, Viral epidemiology

Correction to: *Nature Communications* 10.1038/s41467-021-26707-9, published online 18 November 2021

In this article the VEO grant agreement No. 874735 relating to European Union’s Horizon 2020 research and innovation program was omitted. The original article has been corrected.

