# Supplementary material for: Model-based assessment of Chikungunya and O’nyong-nyong virus circulation in Mali in a serological cross-reactivity context
Source: Nat Commun. 2021 Nov 18;12:6735. doi: 10.1038/s41467-021-26707-9 (PMC8602252; doi:10.1038/s41467-021-26707-9)
Supplement: Supplementary file 1 — Supplementary Information [file 41467_2021_26707_MOESM1_ESM.pdf]

Supplementary Information for

Model-based assessment of Chikungunya and O'nyong-nyong virus circulation in Mali in a serological cross-reactivity context

Nathanaël Hozé, Issa Diarra, Abdoul Karim Sangaré, Boris Pastorino, Laura Pezzi, Bourèma Kouriba, Issaka Sagara, Abdoulaye Dabo, Abdoulaye Djimdé, Mahamadou Ali Thera, Ogobara K. Doumbo, Xavier de Lamballerie, Simon Cauchemez

Email: xavier.de-lamballerie@univ-amu.fr

This file contains Supplementary Figures S1-5 and Supplementary Tables 1-6.

Table S1: Demographic characteristics of the participants in Mali.

Table S2: Supplementary Table 2. Demographic characteristics of the participants in Mali by 10-year age group (from 10- 19 to 60+), and by sampling location.

Table S3: Validation of the statistical framework to estimate parameters.

Table S4: Parameter estimates for the baseline model (Model 1), the model with one-way cross-reactivity where anti-ONNV antibodies never induce a CHIKV response (Model 2), and the model where the cross-reactive response is proportional to the response for the infecting virus (Model 3).

Table S5: Model comparison.

Table S6: Model adequacy.

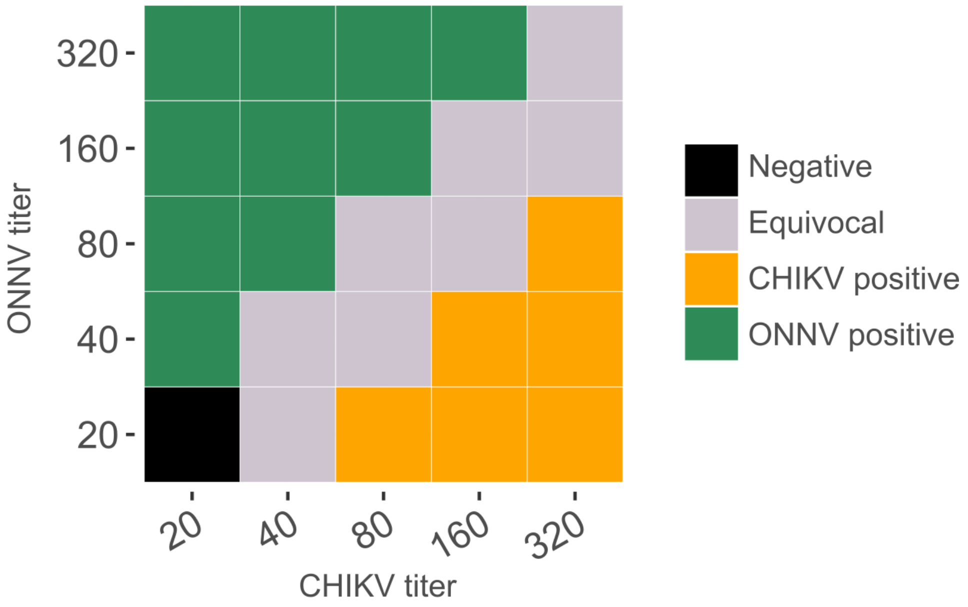

Supplementary Figure 1. Classical method classification of CHIKV and ONNV positive as a function of the paired titers.

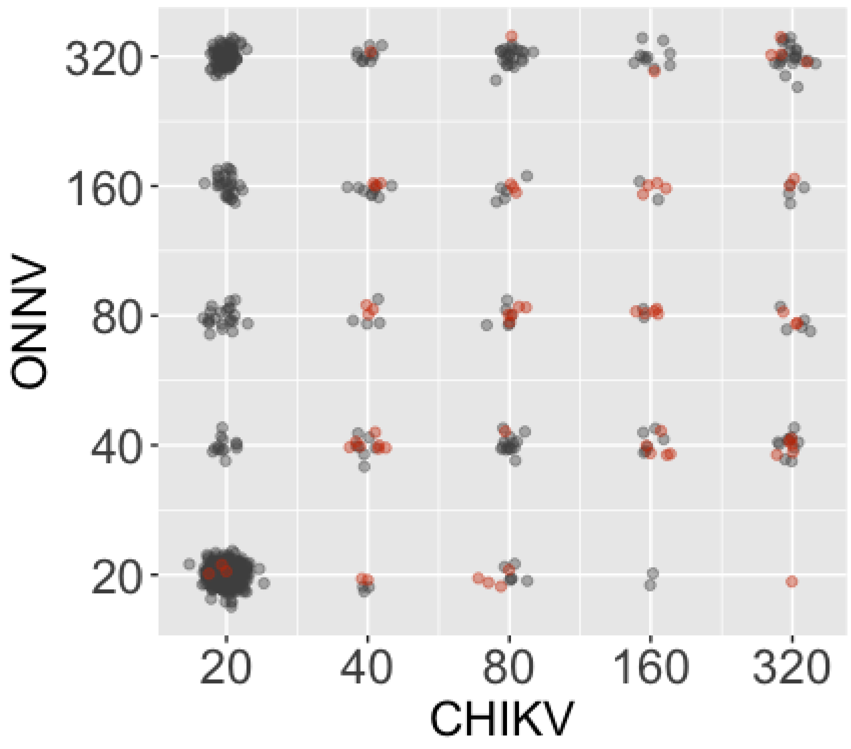

Supplementary Figure 2. Simulated paired ONNV and CHIKV titers in Mali (grey) and Martinique (red), obtained with the median of the posterior parameters.

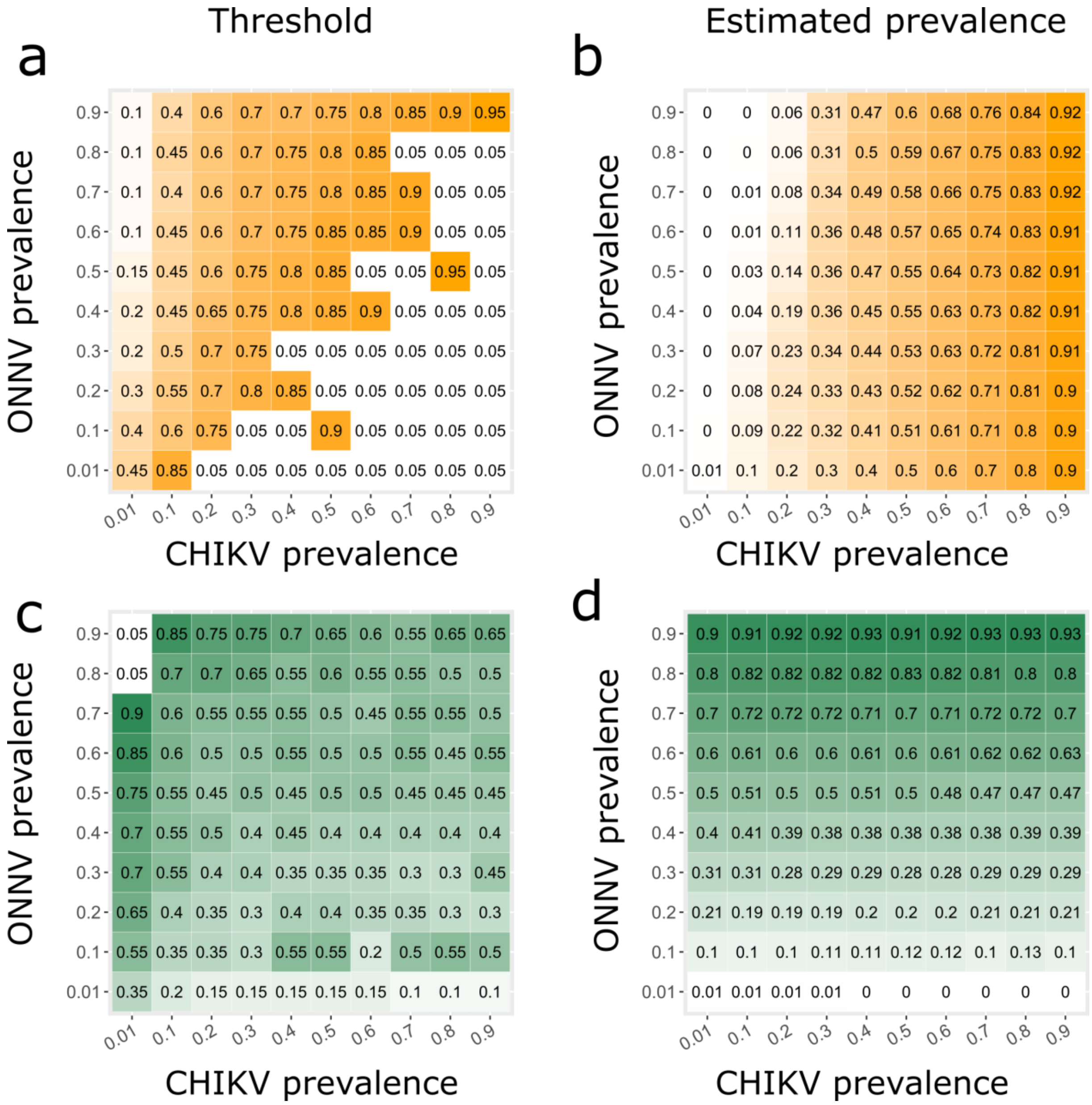

Supplementary Figure 3. Design of the model based-classification using simulated surveys. The probability of infection was estimated for each value of the VNT and compared to a threshold value. The heatmaps show the thresholds of the probability of infection by CHIKV (**a**) and ONNV (**c**) that minimize the difference of the input and estimated prevalence for different values of virus prevalence. (**b,d**) Estimated prevalence for CHIKV (**b**) and ONNV (**d**) obtained at a threshold of 0.65 for CHIKV and 0.5 for ONNV.

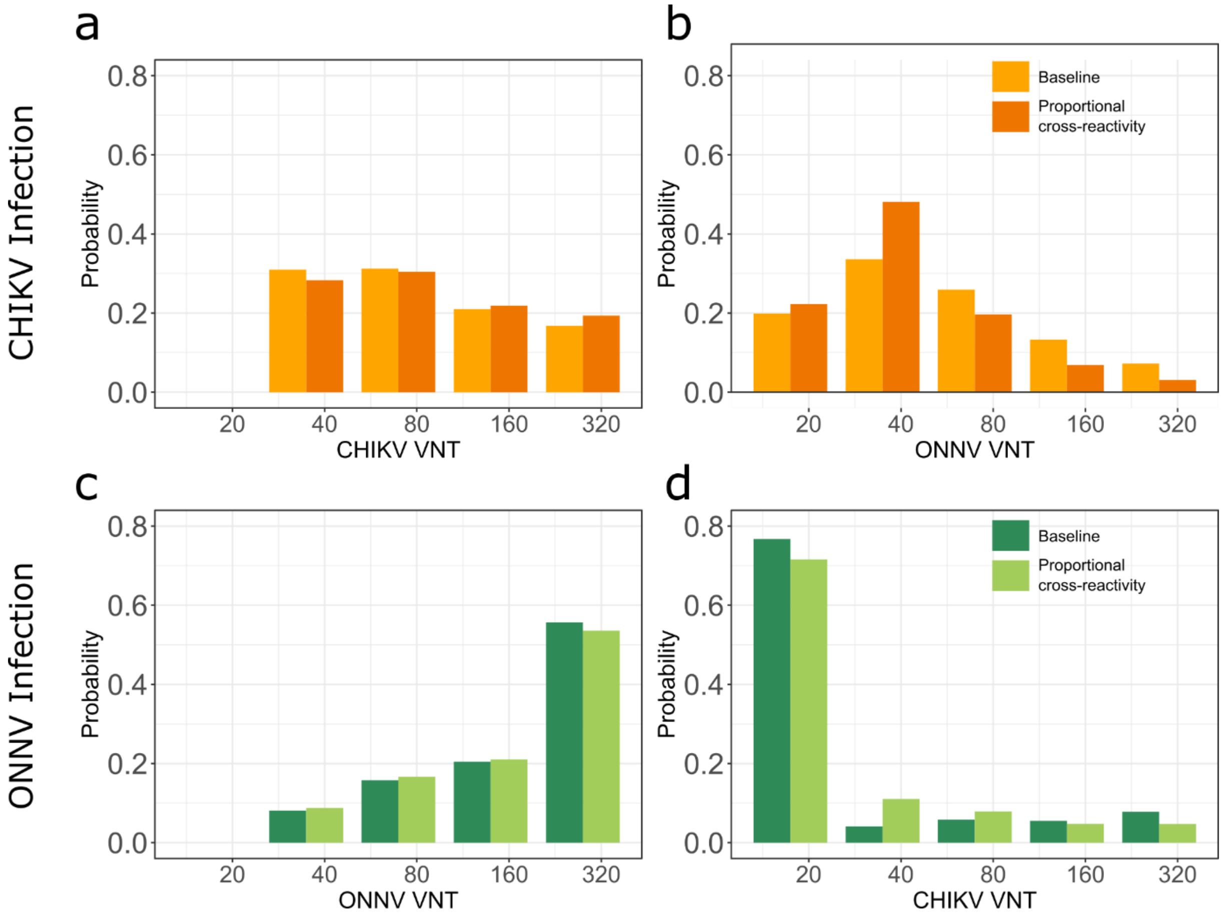

Supplementary Figure 4. Comparison of the response to infection for different models of cross-reactivity. Probability distribution of CHIKV response (**a**, **d**) and ONNV response (**b**,**c**) upon a CHIKV infection (top row) and an ONNV infection (bottow row). The distributions are presented for the baseline model and for the model where the cross-reactive response is proportional to antibody titer boost of the infecting virus.

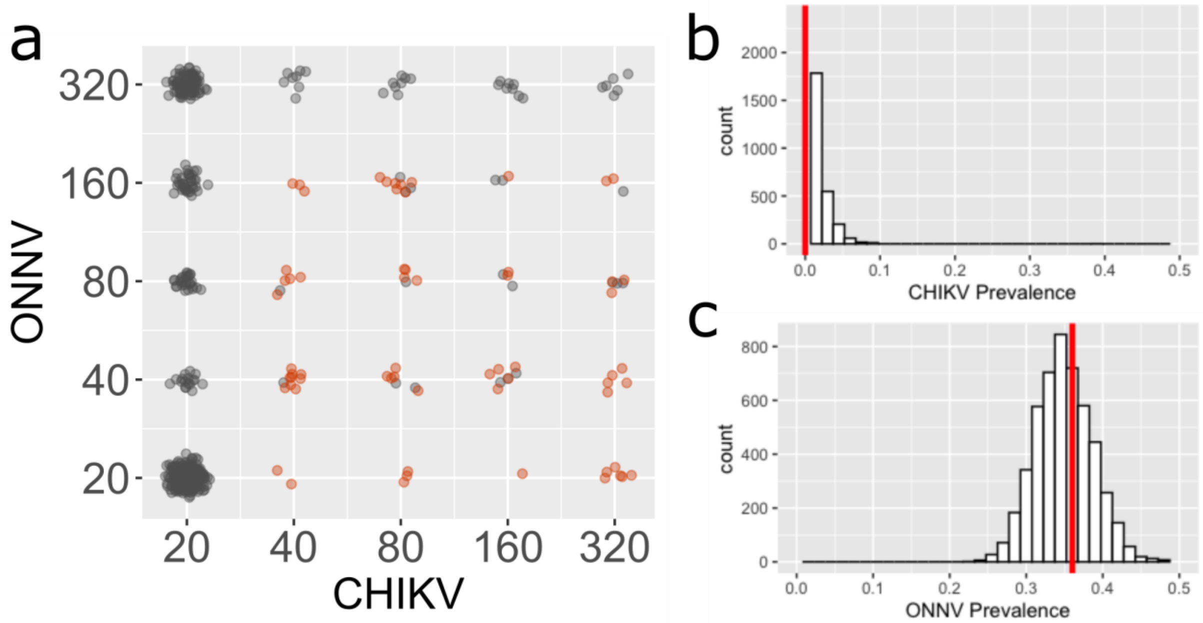

Supplementary Figure 5. Simulated scenario where only ONNV circulated in Mali. (**a**) Paired titers (red: Martinique, gray: Mali). (**b, c**) Posterior distribution of the CHIKV (**b**) and ONNV (**c**) prevalence in Mali. The red vertical line is the input value of the prevalence.

Supplementary Table 1. Demographic characteristics of the participants in Mali.

Sampling site		Participants	Males	Females	Median age, y
North	Diema	109	27	82	28
	Bandiagara	187	58	129	35
	Niono	65	9	56	35
South	Bamako	129	50	79	32
	Kadiolo	136	40	96	30
	Kita	40	16	24	23
	Bougouni	127	42	85	45
Total		793	242	551	33

Supplementary Table 2. Demographic characteristics of the participants in Mali by 10-year age group (from 10- 19 to 60+), and by sampling location.

Region	10 – 19	20 – 29	30 – 39	40 – 49	50 – 59	60+
Diema	19	40	15	8	13	14
Bamako	23	33	22	16	11	24
Kita	14	9	6	2	6	3
Bandiagara	26	46	31	19	26	39
Kadiolo	23	41	27	21	7	17
Niono	17	11	10	8	10	9
Bougouni	12	23	23	11	18	40
Mali (aggregated)	134	203	134	85	91	146

Supplementary Table 3. Validation of the statistical framework to estimate parameters. Input column corresponds to the parameter values used in the simulation study. We report the mean and 95% confidence intervals of the mean posterior from each simulation and the fraction of the simulations for which the input parameters falls into the 95% credible interval of the posterior distribution.

Input	Estimate (mean and 95% confidence interval)	Fraction of times that the input falls in the 95% CrI in the simulations
$\sigma^C = 2.1$	2.1 (1.8 – 2.4)	95%
$\sigma^O = 3.9$	3.8 (3.5 – 4.2)	92%
$\lambda^C = 0.23$	0.23 (0.12 – 0.39)	88%
$\lambda^O = 0.42$	0.45 (0.32 – 0.58)	97%
$p^{C \to O} = 0.81$	0.79 (0.73 – 0.86)	97%
$p^{O \to C} = 0.21$	0.22 (0.09 – 0.33)	94%
$\sigma^{C \to O} = 1.6$	1.6 (1.3 – 2.0)	91%
$\sigma^{O \to C} = 2.6$	3.0 (1.9 – 4.4)	93%
$f_1^C(tropical) = 0.85$	0.92 (0.56 – 1.56)	88%
$f_1^O(tropical) = 0.75$	0.77 (0.61 – 0.98)	97%
$f_2^C(female) = 1.76$	1.96 (1.16 – 2.91)	96%
$f_2^O(female) = 1.09$	1.15 (0.85 – 1.45)	95%

Supplementary Table 4. Parameter estimates for the baseline model (Model 1), the model with one-way cross-reactivity where anti-ONNV antibodies never induce a CHIKV response (Model 2), and the model where the cross-reactive response is proportional to the response for the infecting virus (Model 3). The numbers provided are the mean and 95% credible interval.

Parameter	Description	Model 1	Model 2	Model 3
σ^{C}	CHIKV response to CHIKV infection	2.4 (2.1 – 2.6)	2.5 (2.3 – 2.7)	2.4 (2.2 - 2.7)
σ^{O}	ONNV response to ONNV infection	4.0 (3.6 – 4.3)	3.9 (3.6 – 4.3)	3.9 (3.6 - 4.2)
$\sigma^{C \to O}$	ONNV response to CHIKV infection	2.0 (1.7 – 2.3)	2.3 (2.0 – 2.5)	1.2 (1.2 - 1.3)
$\sigma^{O \to C}$	CHIKV response to ONNV infection	2.9 (2.1 – 4.3)	-	1.3 (1.2 - 1.3)
P^{C}	CHIKV prevalence in Mali	0.13 (0.09 – 0.18)	0.19 (0.16 – 0.22)	0.11 (0.07 - 0.16)
P^{O}	ONNV prevalence in Mali	0.29 (0.25 – 0.34)	0.26 (0.23 – 0.29)	0.31 (0.27 – 0.35)
$p^{C \to O}$	Fraction of CHIKV infected that have a positive ONNV response	0.80 (0.72 – 0.87)	0.84 (0.78 – 0.89)	0.78 (0.68 - 0.85)
$p^{O \to C}$	Fraction of ONNV infected that have a positive CHIKV response	0.22 (0.08 – 0.33)	0	0.29 (0.16 - 0.39)
$f_1^{C}(tropical)$	Risk of CHIKV infection in tropical vs semi-arid regions	0.85 (0.52 – 1.33)	0.87 (0.63 – 1.17)	0.88 (0.48 - 1.51)
$f_1^{O}(tropical)$	Risk of ONNV infection in tropical vs semi-arid regions	0.77 (0.58 – 1.0)	0.74 (0.56 – 0.94)	0.77 (0.59 – 1.0)
$f_2^{C}(female)$	Risk of CHIKV infection for females vs males	1.9 (1.1 – 2.9)	1.8 (1.2 – 2.4)	1.7 (0.81 - 2.9)
$f_2^{O}(female)$	Risk of ONNV infection for females vs males	1.1 (0.80 – 1.5)	1.0 (0.73 – 1.4)	1.2 (0.87 - 1.6)

Supplementary Table 5. Model comparison. Model adequacy was evaluated using the DIC. A smaller DIC indicates a better model adequacy and DIC differences of more than 5 points are considered substantial.

**Model**	**DIC**
Baseline model	3092
Direct response model = Negative binomial	3099
One way-cross-reactivity: Probability of ONNV infection to induce CHIKV response =0	3098
Proportional cross-reactivity model	3089
Age dependent force of infection	3231

Supplementary Table 6. Model adequacy. Comparison of observations (top number) and posterior number of cases in simulated data (bottom, mean and 95% predictive intervals); 2000 surveys were simulated using parameters drawn from the posterior distribution.

ONNV VNT	CHIKV VNT 20	40	80	160	320
320	91<br>86 (64 - 111)	9<br>14 (7 - 23)	13<br>16 (8 - 26)	11<br>12 (6 - 20)	22<br>17 (8 - 27)
160	25<br>32 (21 - 44)	8<br>8 (3 - 14)	8<br>8 (3 - 15)	16<br>6 (2 - 12)	8<br>7 (2 - 13)
180	26<br>25 (14 - 37)	16<br>11 (5 - 19)	11<br>12 (6 - 20)	5<br>9 (3 - 16)	17<br>8 (3 - 15)
40	16<br>13 (6 - 22)	21<br>13 (6 - 22)	8<br>14 (6 - 23)	7<br>10 (4 - 16)	5<br>9 (3 - 16)
20	487<br>500 (462 - 540)	13<br>7 (2 - 14)	11<br>7 (2 - 14)	2<br>5 (1 - 10)	0<br>4 (1 - 10)
