# Supplementary material for: Model-based assessment of Chikungunya and O’nyong-nyong virus circulation in Mali in a serological cross-reactivity context
Source: Nat Commun. 2021 Nov 18;12:6735. doi: 10.1038/s41467-021-26707-9 (PMC8602252; doi:10.1038/s41467-021-26707-9)
Supplement: Supplementary file 3 — Reporting Summary [file 41467_2021_26707_MOESM3_ESM.pdf]

nature research

Corresponding author(s): Xavier de Lamballerie

Last updated by author(s): Sep 1, 2021

Reporting Summary

Nature Research wishes to improve the reproducibility of the work that we publish. This form provides structure for consistency and transparency in reporting. For further information on Nature Research policies, see our Editorial Policies and the Editorial Policy Checklist.

Statistics

For all statistical analyses, confirm that the following items are present in the figure legend, table legend, main text, or Methods section.

n/a	Confirmed	
☐	☒	The exact sample size (*n*) for each experimental group/condition, given as a discrete number and unit of measurement
☐	☒	A statement on whether measurements were taken from distinct samples or whether the same sample was measured repeatedly
☒	☐	The statistical test(s) used AND whether they are one- or two-sided<br>*Only common tests should be described solely by name; describe more complex techniques in the Methods section.*
☐	☒	A description of all covariates tested
☒	☐	A description of any assumptions or corrections, such as tests of normality and adjustment for multiple comparisons
☐	☒	A full description of the statistical parameters including central tendency (e.g. means) or other basic estimates (e.g. regression coefficient) AND variation (e.g. standard deviation) or associated estimates of uncertainty (e.g. confidence intervals)
☒	☐	For null hypothesis testing, the test statistic (e.g. *F*, *t*, *r*) with confidence intervals, effect sizes, degrees of freedom and *P* value noted<br>*Give P values as exact values whenever suitable.*
☐	☒	For Bayesian analysis, information on the choice of priors and Markov chain Monte Carlo settings
☐	☒	For hierarchical and complex designs, identification of the appropriate level for tests and full reporting of outcomes
☒	☐	Estimates of effect sizes (e.g. Cohen's *d*, Pearson's *r*), indicating how they were calculated

Our web collection on statistics for biologists contains articles on many of the points above.

Software and code

Policy information about availability of computer code

Data collection	Data were collected using tablets and the Open Data Kit (ODK) platform system.
Data analysis	Custom code was developed in R (version 3.6.1). MCMC algorithm was performed using the R package rstan (version 2.19.2). The code is available from the GitHub link at https://github.com/nathoze/ONNV_CHIKV

For manuscripts utilizing custom algorithms or software that are central to the research but not yet described in published literature, software must be made available to editors and reviewers. We strongly encourage code deposition in a community repository (e.g. GitHub). See the Nature Research guidelines for submitting code & software for further information.

Data

Policy information about availability of data

All manuscripts must include a data availability statement. This statement should provide the following information, where applicable:

- Accession codes, unique identifiers, or web links for publicly available datasets
- A list of figures that have associated raw data
- A description of any restrictions on data availability

The following data is available in a format that maintains anonymity of survey participants from the GitHub link at https://github.com/nathoze/ONNV_CHIKV. For each individual: age group (10-year classes), ONNV VNT, CHIKV VNT, region (North or South), sex.

Field-specific reporting

Please select the one below that is the best fit for your research. If you are not sure, read the appropriate sections before making your selection.

☐ Life sciences ☒ Behavioural & social sciences ☐ Ecological, evolutionary & environmental sciences

For a reference copy of the document with all sections, see nature.com/documents/nr-reporting-summary-flat.pdf

Behavioural & social sciences study design

All studies must disclose on these points even when the disclosure is negative.

Study description	Cross-sectional study primarily dedicated to establishing the seroprevalence of arboviral and haemorrhagic viral infections in Mali
Research sample	sera from 793 volunteers (over 14 years, 242 males and 551 females) in seven sampling sites in Mali. The study was preliminarily a cross-sectional study dedicated to establishing the seroprevalence of arboviral and haemorrhagic viral infections in Mali.
Sampling strategy	Sample size and sampling strategy: Villages or city areas were randomly selected from an exhaustive list in each district. Families were randomly selected and volunteers were recruited with the objective of including 100-150 individuals per site, which corresponds to the estimate of the recruitment capacity by field teams for each site investigated. This sample size allows establishing seroprevalence in each site and globally.<br><br>Study sites: We selected seven districts representing the different eco-climatic areas of Mali: Diema (109), Kita (40), Bougouni (127), Kadiolo (136), Niono (65), Bandiagara (187) and Commune IV of Bamako (129). Those districts are spread over the different administrative regions of Mali - except the northern region, which could not be investigated for security reasons. The selected districts are also those used by the Ministry of Health for infectious diseases surveillance.
Data collection	Enrolment and data collection: Only healthy, non febrile, volunteers (males and females) aged ≥15 years old (yo) were enrolled. Information was provided in the most familiar language of the volunteer, and in the presence of an observer designed by the village authorities for ensuring a complete understanding by the volunteer and helping obtaining answers to any question. All volunteers signed the Informed Consent form prior to enrolment (and received a copy of the signed form). Data were collected using tablets and the Open Data Kit (ODK) platform system. This included in the current study socio-demographic information, weight, and history of travels. The researcher was not blinded, since all relevant samples were tested for a seroprevalence study.
Timing	Between October and November 2016
Data exclusions	The northern region of Mali was excluded because it could not be investigated for security reasons.
Non-participation	No participants dropped out
Randomization	our study was a cross sectional study during which villages/city areas and families were randomly selected from an exhaustive list in each district and volunteers included in arrival order from randomlized family; this allows us to avoid the bias for demographic covariates.

Reporting for specific materials, systems and methods

We require information from authors about some types of materials, experimental systems and methods used in many studies. Here, indicate whether each material, system or method listed is relevant to your study. If you are not sure if a list item applies to your research, read the appropriate section before selecting a response.

Materials & experimental systems

n/a	Involved in the study	
☐	☒	Antibodies
☐	☒	Eukaryotic cell lines
☒	☐	Palaeontology and archaeology
☒	☐	Animals and other organisms
☐	☒	Human research participants
☒	☐	Clinical data
☒	☐	Dual use research of concern

Methods

n/a	Involved in the study	
☒	☐	ChIP-seq
☒	☐	Flow cytometry
☒	☐	MRI-based neuroimaging

Antibodies

Antibodies used	we used Euroimmun anti-NS1 IgG ELISA kit, from Medizinische Labordiagnostika (Lübeck, Germany) to detect Anti-CHIKV IgG antibodies.

Validation	Sensibility and specificity values of this test were 95.4% and 100% respectively (Nadine Litzba and al., Journal of Virological Methods ; 2008. https://doi.org/10.1016/j.jviromet.2008.01.004)

Eukaryotic cell lines

Policy information about cell lines

Cell line source(s)	Vero cell line CCL-81 (cells from kidney of african green monkey), purchased from American Type Cell Collection (ATCC) at Manassas, Virginia, USA.
Authentication	American Type Cell Collection (ATCC) at Manassas, Virginia, USA.
Mycoplasma contamination	No mycoplasma contamination
Commonly misidentified lines (See ICLAC register)	no commonly misidentified cell lines were used in the study

Human research participants

Policy information about studies involving human research participants

Population characteristics	793 volunteers aged ≥15 years old (yo) from cross sectional study in 7 Malian localities. sex ratio (M/F) was 0.44 (242/551), median age was 33 years.
Recruitment	Only healthy, (non febrile, males and females) were enrolled. Volunteer was included in arrival order from randomized families. Potential recruitment biases: sample size (which did not represent Malian general population); exclusion of warm desert regions (difficult access because of military conflicts); exclusion of children under 15 years and patients with fever.
Ethics oversight	Ethics statement: First, we obtained the approbation of The Institutional Review Board of the Faculty of Medicine and Odonto-Stomatology, University of Sciences, Techniques and Technologies, Bamako, Mali (IRB letter no. 2016/113/CE/FMPOS), and then we visited all sites to explain the study context to health professionals, administrative authorities, and local community. Villages or city areas were randomly selected from an exhaustive list in each district. After obtain community permission, volunteers were recruited from families randomly selected. Participant received two days before their inclusion the study information and the form for informed consent in his/her family language in the presence of a witness designated by the village authorities. For participants aged under 18 years, we obtained both their informed assents and parent/guardian informed consent. Volunteers and witnesses signed two informed consent forms before enrolment; a signed copy was given to participant. We conducted the study according to institutional procedures and guidelines.

Note that full information on the approval of the study protocol must also be provided in the manuscript.
